# Supplementary material for: A genome-scale drug discovery pipeline uncovers new therapeutic targets and a unique p97 allosteric binding site in Schistosoma mansoni
Source: bioRxiv. 2025 Mar 15:2025.03.14.643303. Preprint. [Version 1] doi: 10.1101/2025.03.14.643303 (PMC11952559; doi:10.1101/2025.03.14.643303)
Supplement: 1 [file NIHPP2025.03.14.643303V1-supplement-1.pdf]

bioRxiv preprint doi: https://doi.org/10.1101/2025.03.14.643303; this version posted March 15, 2025. The copyright holder for this preprint (which was not certified by peer review) is the author/funder, who has granted bioRxiv a license to display the preprint in perpetuity. It is made available under aCC-BY 4.0 International license.

Supplemental Figure 1.

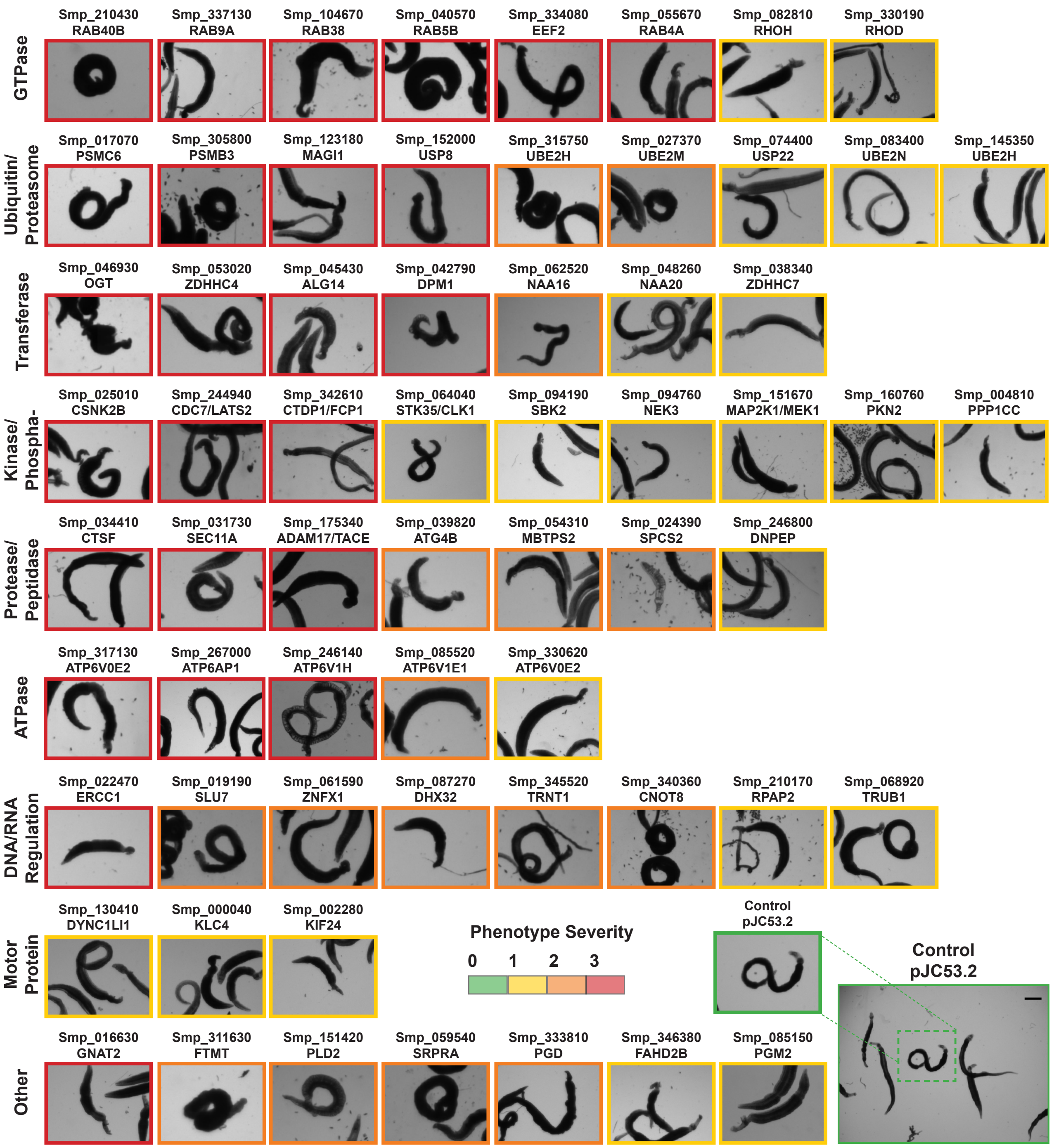

bioRxiv preprint doi: https://doi.org/10.1101/2025.03.14.643303; this version posted March 15, 2025. The copyright holder for this preprint (which was not certified by peer review) is the author/funder, who has granted bioRxiv a license to display the preprint in perpetuity. It is made available under aCC-BY 4.0 International license.

Supplemental Figure 1. Treatment of adult parasites with dsRNA targeting potential druggable genes

Light microscopy images of adult parasites treated with control dsRNA (pJC53.2) and dsRNAs targeting potential druggable genes within *S. mansoni* that bear homology to human drug targets. Targets were arranged according to order found in Figure 1c of enzymatic activity classification and phenotype severity. Scale bar, 1,000 µm.

bioRxiv preprint doi: https://doi.org/10.1101/2025.03.14.643303; this version posted March 15, 2025. The copyright holder for this preprint (which was not certified by peer review) is the author/funder, who has granted bioRxiv a license to display the preprint in perpetuity. It is made available under aCC-BY 4.0 International license.

395 **Supplemental Figure 2.**

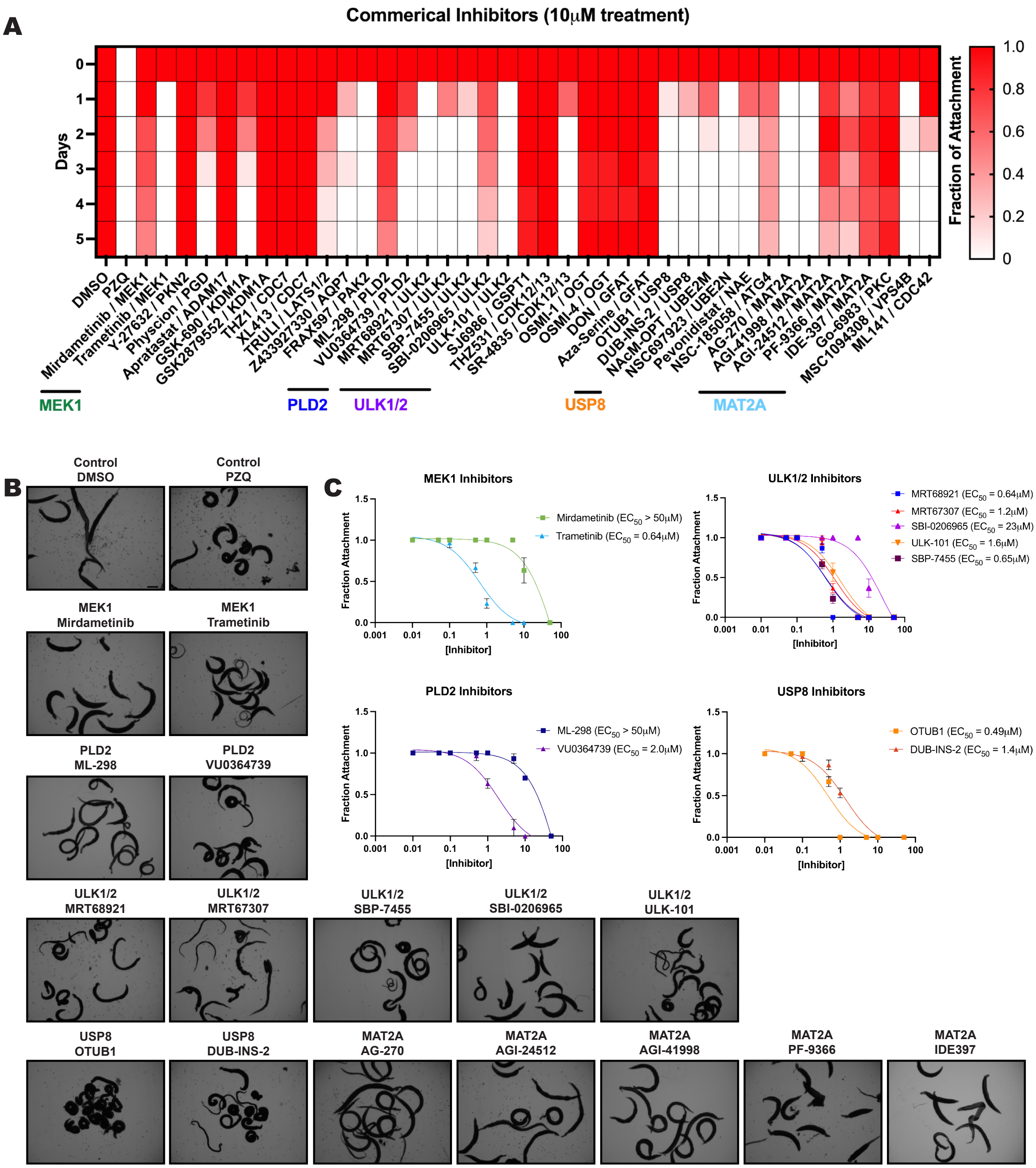

396

bioRxiv preprint doi: https://doi.org/10.1101/2025.03.14.643303; this version posted March 15, 2025. The copyright holder for this preprint (which was not certified by peer review) is the author/funder, who has granted bioRxiv a license to display the preprint in perpetuity. It is made available under aCC-BY 4.0 International license.

Supplemental Figure 2. Treatment of worms with human drug target inhibitors

(A) Heat map showing time course following treatment of adult worms at 10 μM with commercially available inhibitors targeting human orthologs of essential genes identified in RNAi experiments (DMSO; negative control, PZQ; positive control). The fraction of a population of 10 adult worms attached to the culture plate is quantified (dark red; 1 - complete attachment of entire population, white; 0 - no attachment of any worms in population). Worms were treated with inhibitor for 72 hours, replacing drug and media every 24 hr, then monitored until the end of the experiment on day 5. **(B)** Light microscopy images of worms treated with DMSO control or reported inhibitors of human orthologs of essential schistosome genes; MEK1, PLD2, ULK1/2, USP8, and sMAT2A. **(C)** Dose-response curves of worms treated with inhibitors targeting human PLD2, MEK1, ULK1/2, and USP8. Compounds were tested from a range of 50 μM to 10 nM to determine EC_{50}. Values were determined by Prism. Scale bar **(B)**, 1,000 μm.

bioRxiv preprint doi: https://doi.org/10.1101/2025.03.14.643303; this version posted March 15, 2025. The copyright holder for this preprint (which was not certified by peer review) is the author/funder, who has granted bioRxiv a license to display the preprint in perpetuity. It is made available under aCC-BY 4.0 International license.

Supplemental Figure 3.

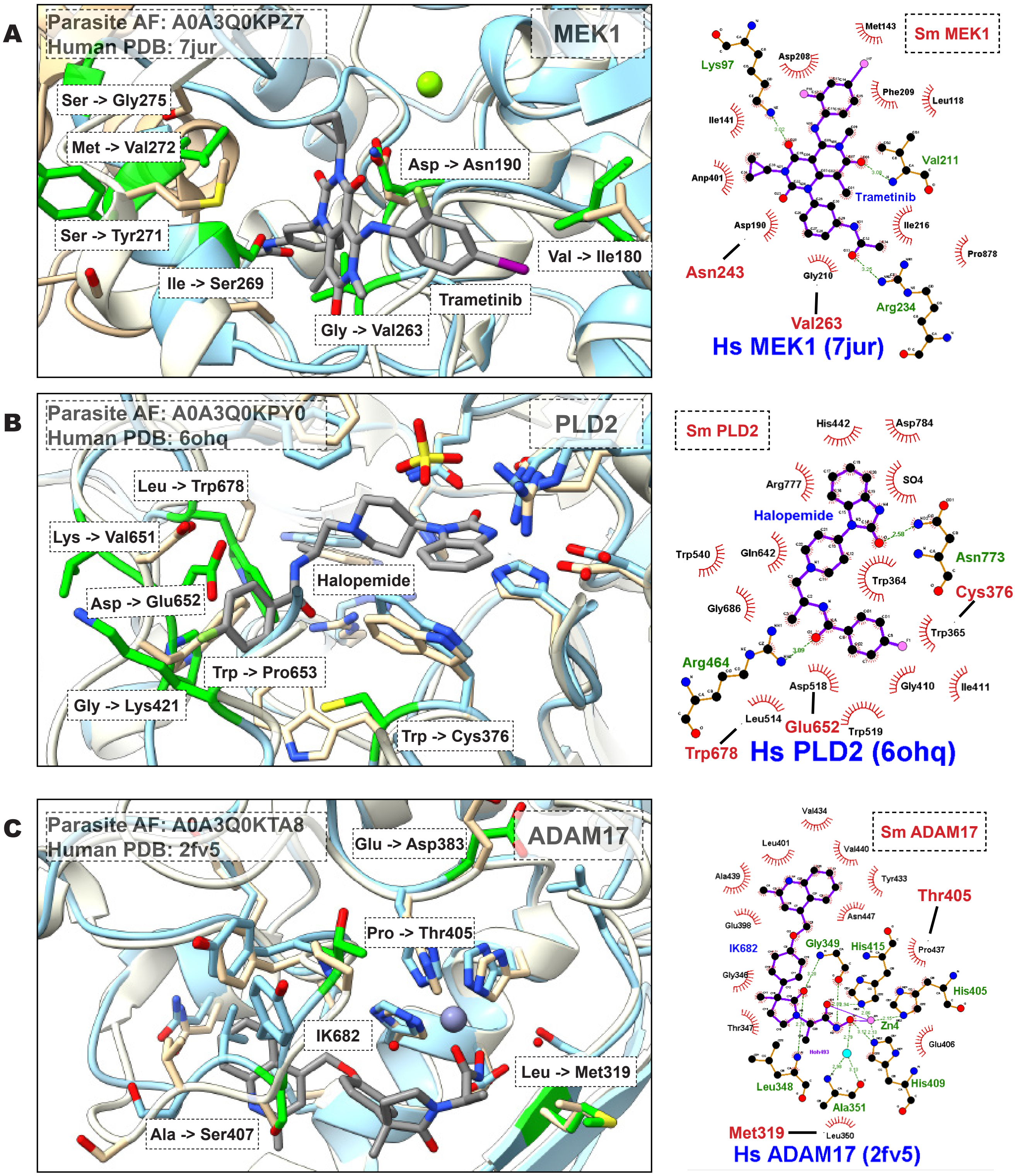

bioRxiv preprint doi: https://doi.org/10.1101/2025.03.14.643303; this version posted March 15, 2025. The copyright holder for this preprint (which was not certified by peer review) is the author/funder, who has granted bioRxiv a license to display the preprint in perpetuity. It is made available under aCC-BY 4.0 International license.

Supplemental Figure 3. 3D homology models of potential schistosome drug targets and their human orthologs

3D alignments of predicted AlphaFold structures of schistosome (blue) proteins overlayed with structures of their closest human (white) homologs. Structures for human proteins bound to their respective inhibitors were retrieved from the Protein Data Bank for **(A)** MEK1 (PDB: 7JUR), **(B)** PLD2 (PDB: 6OHQ), and **(C)** ADAM17 (PDB: 2FV5). Unique schistosome residues are highlighted with green. Ligplots were generated using LigPlot+ v2.2 using the same PDB structures for human orthologs as listed above. Residues forming hydrophobic interactions are colored black, while other interactions are depicted in green. Unique schistosome residues are outlined in red.

bioRxiv preprint doi: https://doi.org/10.1101/2025.03.14.643303; this version posted March 15, 2025. The copyright holder for this preprint (which was not certified by peer review) is the author/funder, who has granted bioRxiv a license to display the preprint in perpetuity. It is made available under aCC-BY 4.0 International license.

420 **Supplemental Figure 4.**

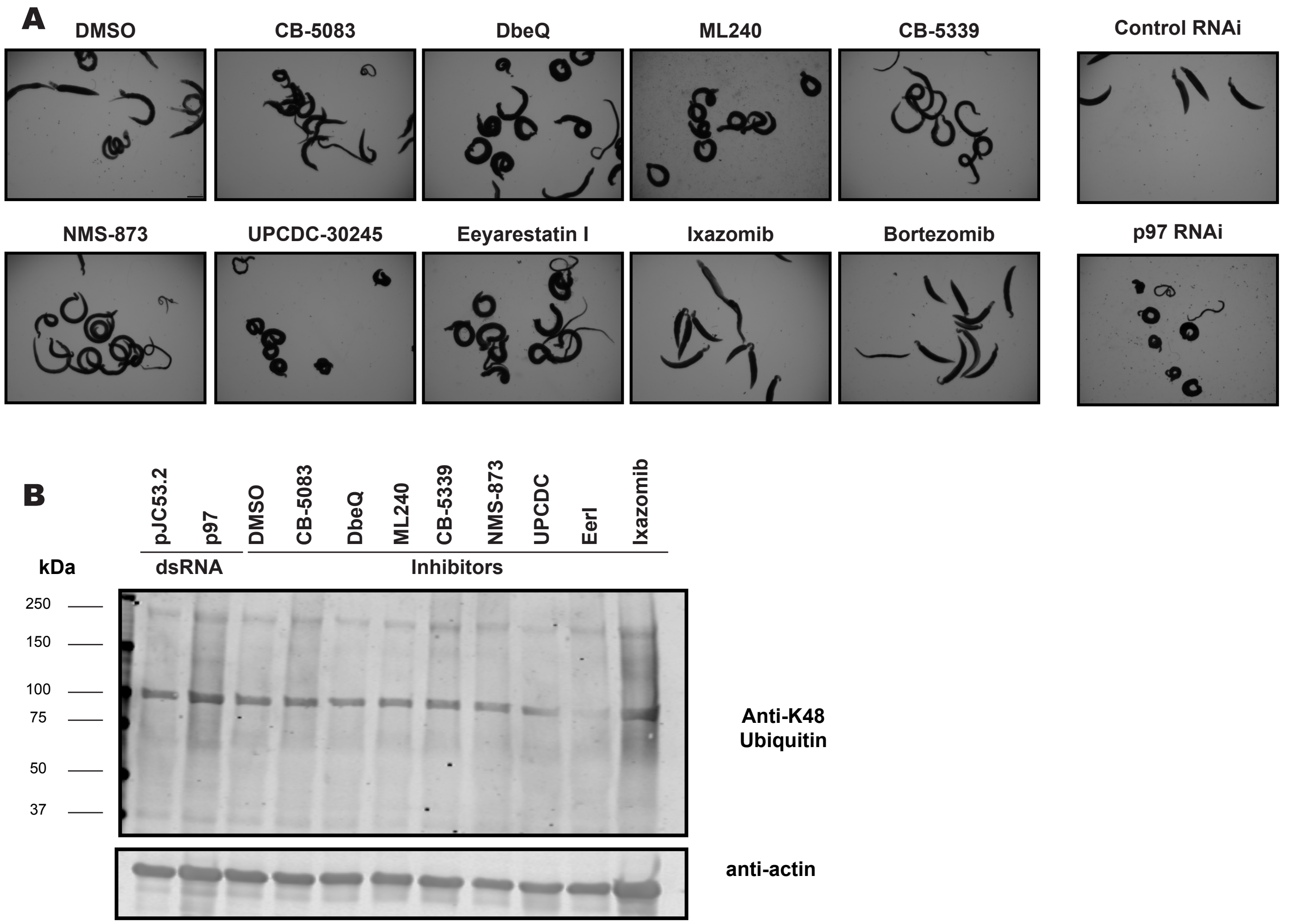

421

bioRxiv preprint doi: https://doi.org/10.1101/2025.03.14.643303; this version posted March 15, 2025. The copyright holder for this preprint (which was not certified by peer review) is the author/funder, who has granted bioRxiv a license to display the preprint in perpetuity. It is made available under aCC-BY 4.0 International license.

Supplemental Figure 4. Treatment of adult parasites with known human p97 inhibitors

(A) Light microscopy images of adult parasites treated with either dsRNA targeting p97 (pJC53.2 control) or human p97 inhibitors (DMSO control) at 10 μM. **(B)** Western blot depicting polyubiquitinated protein profile (K48 antibody) in worm lysate following treatment of adult worms with p97 dsRNA or inhibitors (actin loading control). Scale bar **(A)**, 1,000 μm.

bioRxiv preprint doi: https://doi.org/10.1101/2025.03.14.643303; this version posted March 15, 2025. The copyright holder for this preprint (which was not certified by peer review) is the author/funder, who has granted bioRxiv a license to display the preprint in perpetuity. It is made available under aCC-BY 4.0 International license.

428 **Supplemental Figure 5.**

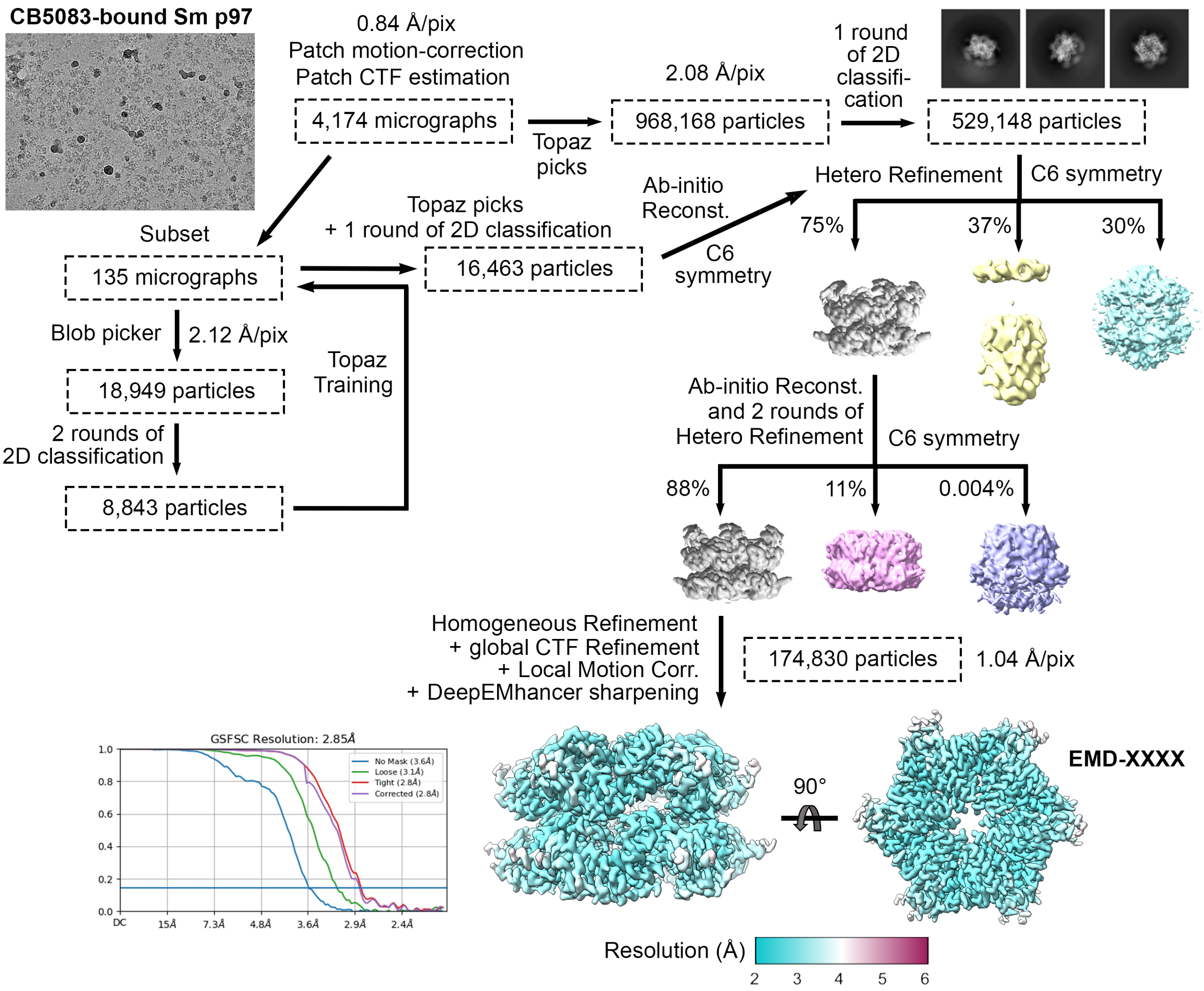

429

bioRxiv preprint doi: https://doi.org/10.1101/2025.03.14.643303; this version posted March 15, 2025. The copyright holder for this preprint (which was not certified by peer review) is the author/funder, who has granted bioRxiv a license to display the preprint in perpetuity. It is made available under aCC-BY 4.0 International license.

Supplemental Figure 5. Data processing of *S. mansoni* p97 bound to CB-5083

Data processing scheme for cryo-EM dataset involving *S. mansoni* p97 bound to active site inhibitor CB-5083.

bioRxiv preprint doi: https://doi.org/10.1101/2025.03.14.643303; this version posted March 15, 2025. The copyright holder for this preprint (which was not certified by peer review) is the author/funder, who has granted bioRxiv a license to display the preprint in perpetuity. It is made available under aCC-BY 4.0 International license.

Supplemental Figure 6.

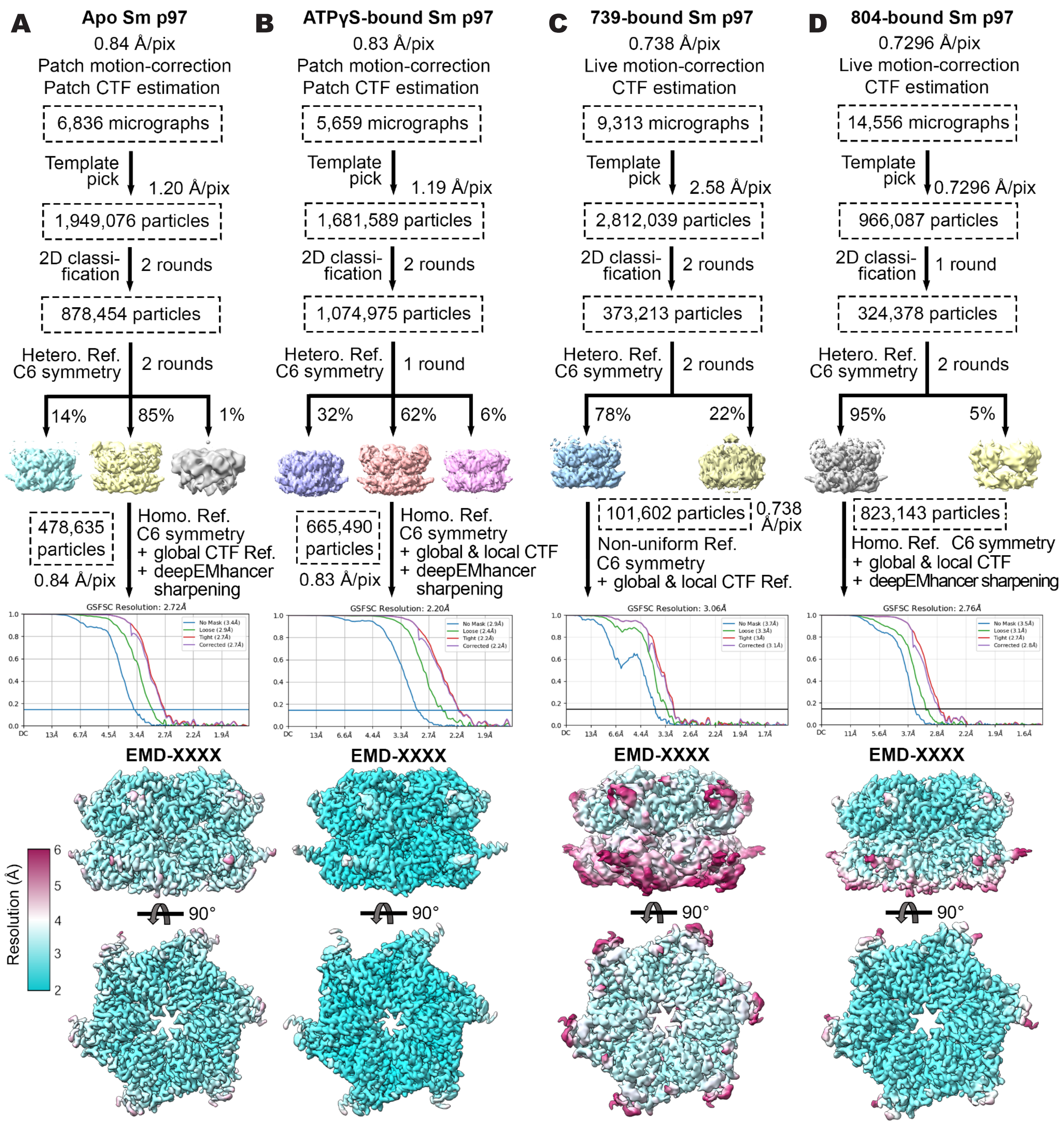

bioRxiv preprint doi: https://doi.org/10.1101/2025.03.14.643303; this version posted March 15, 2025. The copyright holder for this preprint (which was not certified by peer review) is the author/funder, who has granted bioRxiv a license to display the preprint in perpetuity. It is made available under aCC-BY 4.0 International license.

Supplemental Figure 6. Data processing of *S. mansoni* p97 apo enzyme, and bound to ATPγS and covalent inhibitor analogs 739 and 804

(A-D) Data processing scheme for cryo-EM datasets involving *S. mansoni* p97 **(A)** apo enzyme and bound to **(B)** ATPγS and covalent inhibitors, **(C)** 739 and **(D)** 804.

bioRxiv preprint doi: https://doi.org/10.1101/2025.03.14.643303; this version posted March 15, 2025. The copyright holder for this preprint (which was not certified by peer review) is the author/funder, who has granted bioRxiv a license to display the preprint in perpetuity. It is made available under aCC-BY 4.0 International license.

Supplemental Figure 7.

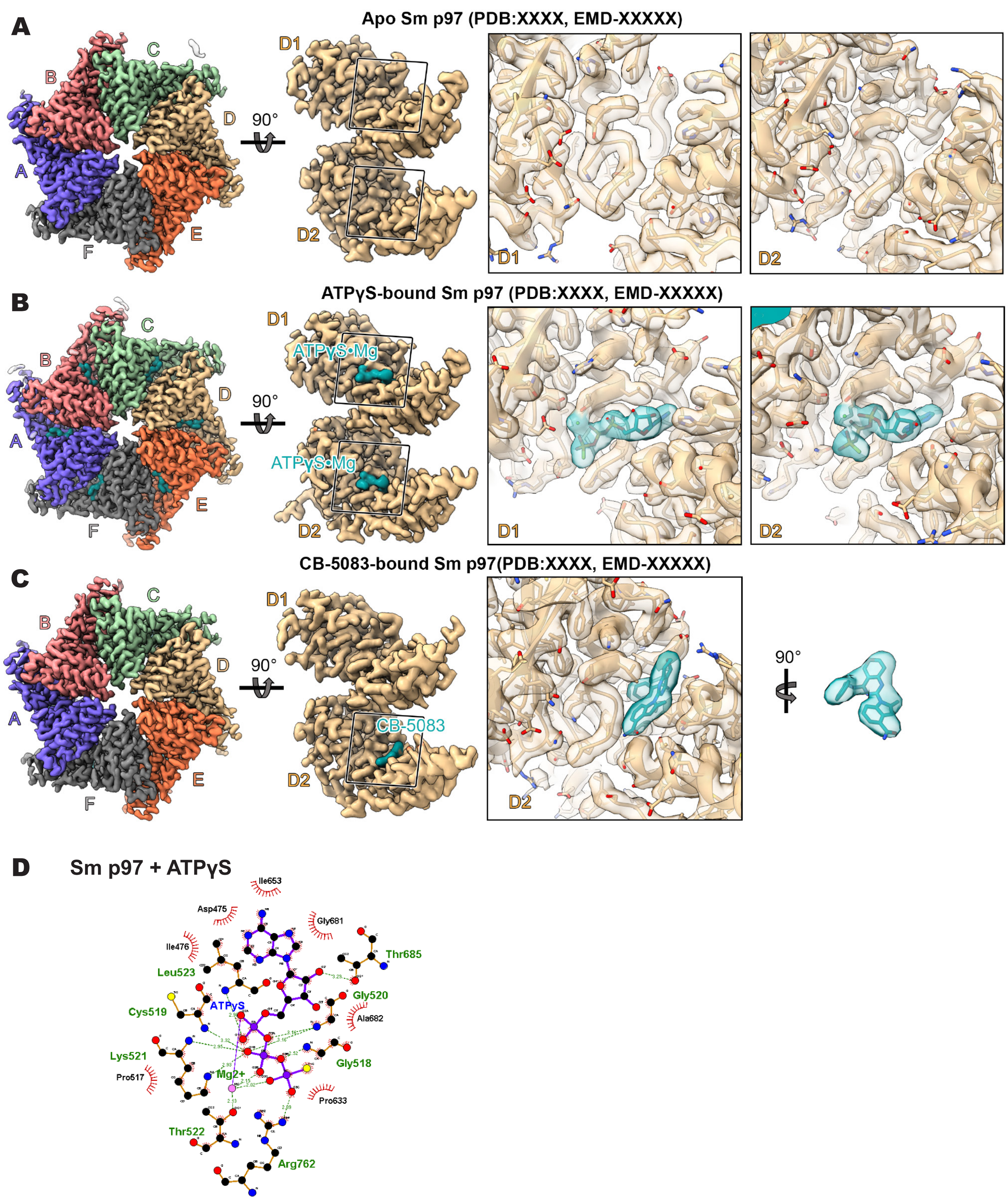

bioRxiv preprint doi: https://doi.org/10.1101/2025.03.14.643303; this version posted March 15, 2025. The copyright holder for this preprint (which was not certified by peer review) is the author/funder, who has granted bioRxiv a license to display the preprint in perpetuity. It is made available under aCC-BY 4.0 International license.

Supplemental Figure 7. Cryo-EM map and structure of *S. mansoni* p97 apo enzyme and bound to known ligands, ATPγS and CB-5083

(A-C) Cryo-EM map of hexamer of schistosome p97 **(A)** apo enzyme or bound to **(B)** ATPγS or **(C)** CB-5083, colored by the final structure. Zoom of ATP binding pocket in the D1 and D2 domain of apo *S. mansoni* p97 (chain D). Boxes on the right show the map quality of the two nucleotide binding pockets. Density for CB-5083 is shown in two views. **(D)** Ligplot of residues involved in *S. mansoni* p97 binding to ATPγS in the D2 domain. Residues forming hydrophobic interactions are colored black, while other interactions are depicted in green.

bioRxiv preprint doi: https://doi.org/10.1101/2025.03.14.643303; this version posted March 15, 2025. The copyright holder for this preprint (which was not certified by peer review) is the author/funder, who has granted bioRxiv a license to display the preprint in perpetuity. It is made available under aCC-BY 4.0 International license.

451 **Supplemental Figure 8.**

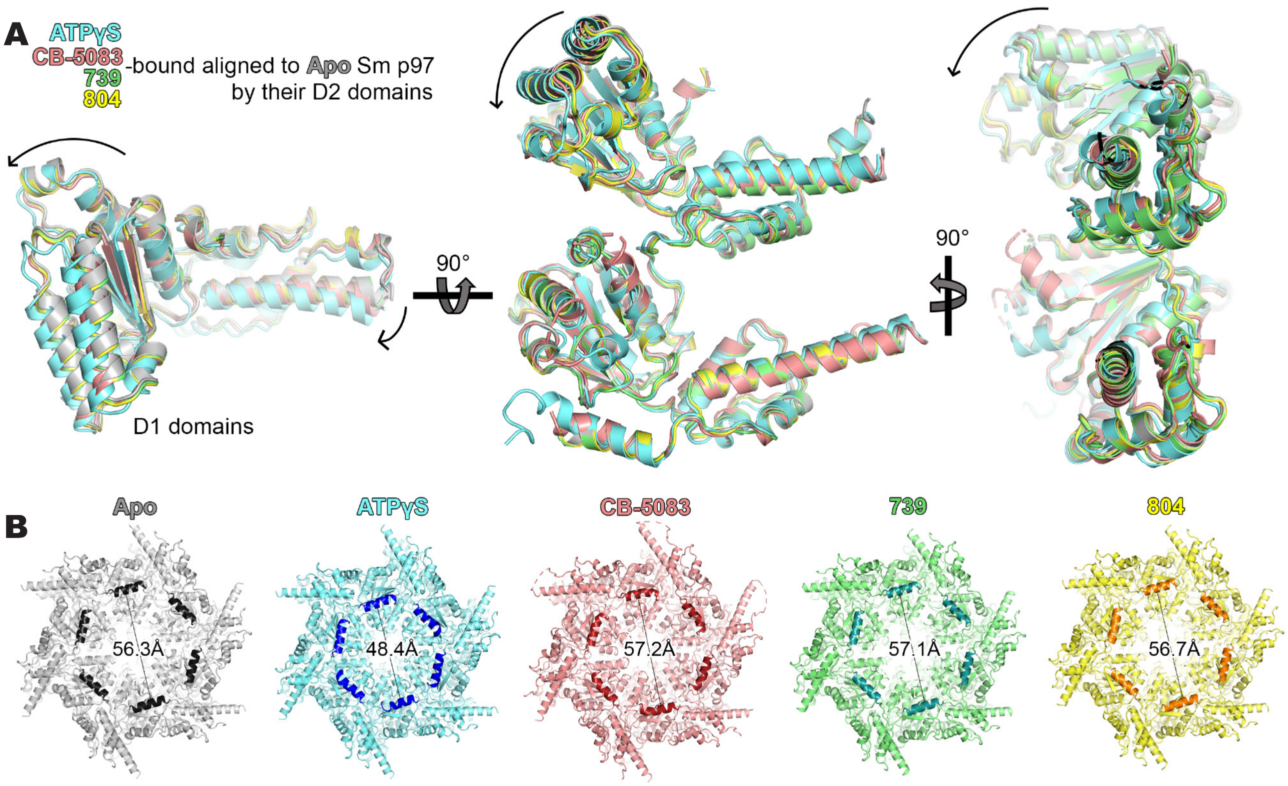

452

bioRxiv preprint doi: https://doi.org/10.1101/2025.03.14.643303; this version posted March 15, 2025. The copyright holder for this preprint (which was not certified by peer review) is the author/funder, who has granted bioRxiv a license to display the preprint in perpetuity. It is made available under aCC-BY 4.0 International license.

Supplemental Figure 8. Conformational changes induced in *S. mansoni* p97 following ligand binding

Depiction of schistosome p97 conformational changes following binding to nucleotide substrate (ATPγS) and small molecule inhibitors (CB-5083, 739, 804) **(A)** Overlay of *S. mansoni* D1 domains following alignment to apo-enzyme D2 domain. **(B)** Bottom-up view of the *S. mansoni* p97 hexamer. Measurement of the diameter of the central pore of the schistosome p97 between residue K750 of two opposite chains in helix 750-757 (bold) for apo enzyme (grey) compared to ligand-bound states (blue; ATPγS, pink; CB-5083, green; 739, yellow; 804).

bioRxiv preprint doi: https://doi.org/10.1101/2025.03.14.643303; this version posted March 15, 2025. The copyright holder for this preprint (which was not certified by peer review) is the author/funder, who has granted bioRxiv a license to display the preprint in perpetuity. It is made available under aCC-BY 4.0 International license.

461 **Supplemental Figure 9.**

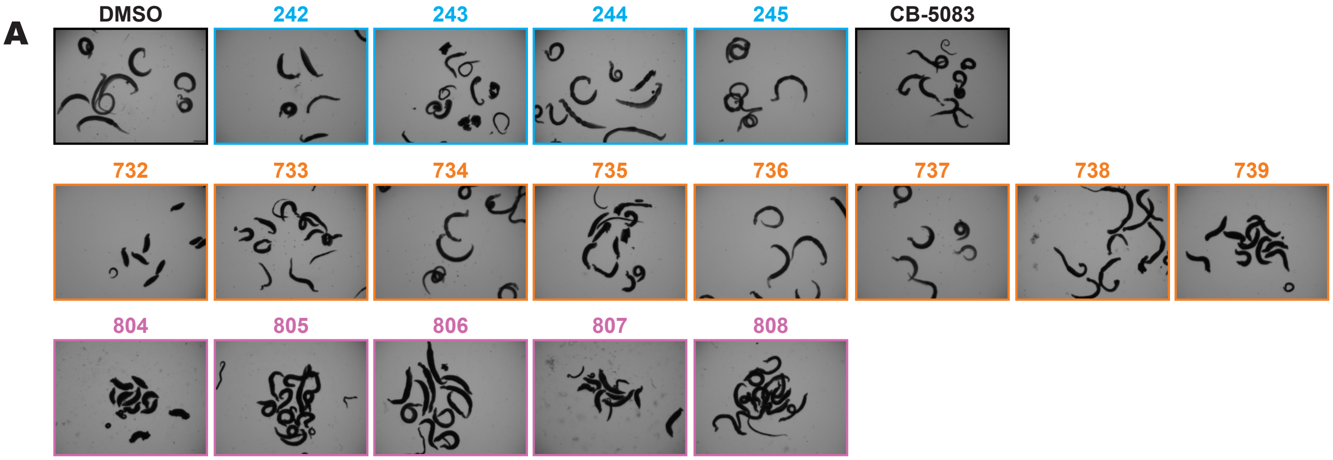

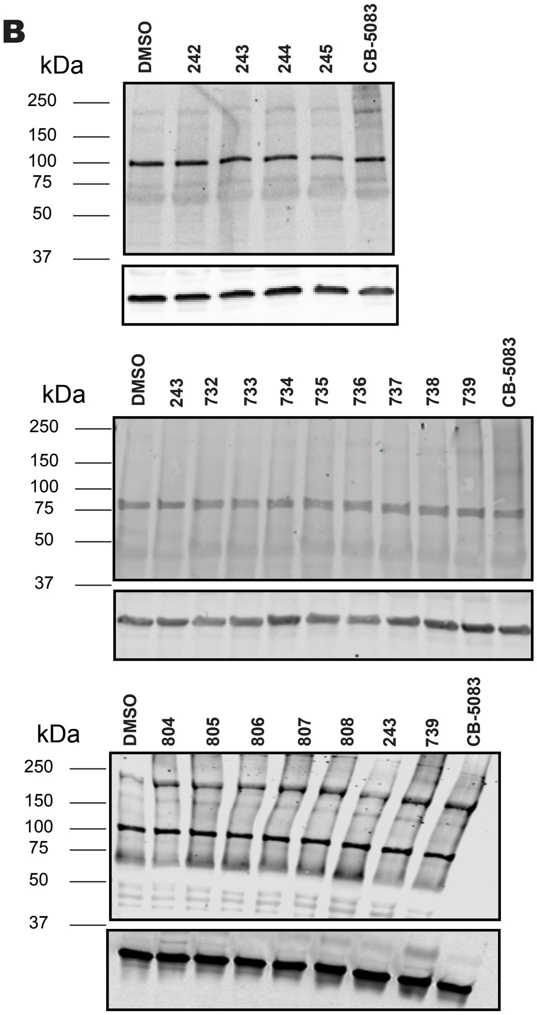

C

Compound	R1	R2	*S. mansoni* IC_{50} (μM)	*H. sapiens* IC_{50} (μM)	Fold Selectivity	S. mansoni EC_{50} (μM)
243	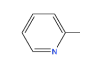	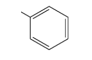	2.1 ± 0.75	20 ± 0.54	9.5	3.5
732	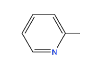	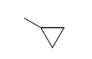	>100	NA	NA	1.3
733	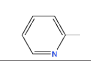	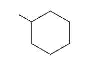	34 ± 1.6	43 ± 4.4	1.3	3.2
734	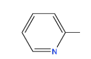	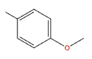	48 ± 2.6	83 ± 6.5	1.7	7.0
735	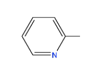	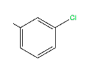	11 ± 0.47	7.3 ± 0.66	0.70	4.2
736	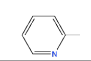	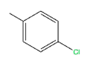	5.9 ± 1.3	6.7 ± 0.055	1.1	12
737	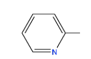	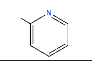	3.0 ± 0.41	13 ± 2.2	4.3	22
738	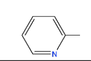	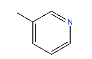	0.42 ± 0.057	0.78 ± 0.14	1.9	3.5
739	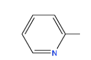	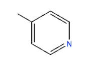	0.15 ± 0.049	0.32 ± 0.026	2.1	1.3
804	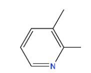	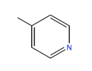	0.19 ± 0.063	0.14 ± 0.062	0.70	0.76
805	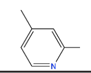	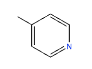	0.17 ± 0.092	0.14 ± 0.078	0.80	2.0
806	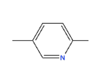	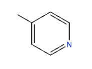	0.47 ± 0.17	0.51 ± 0.16	1.1	3.2
807	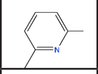	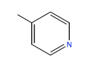	0.67 ± 0.17	0.49 ± 0.057	0.70	1.8
808	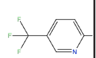	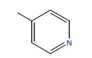	0.18 ± 0.026	0.41 ± 0.084	2.3	2.4

462

bioRxiv preprint doi: https://doi.org/10.1101/2025.03.14.643303; this version posted March 15, 2025. The copyright holder for this preprint (which was not certified by peer review) is the author/funder, who has granted bioRxiv a license to display the preprint in perpetuity. It is made available under aCC-BY 4.0 International license.

Supplemental Figure 9. Treatment of adult parasites with analogs of covalent p97 scaffold identified in high-throughput screen and known human p97 inhibitors

(A) Light microscopy images of adult parasites treated with controls (negative; DMSO, positive; CB-5083) and analogs of the covalent p97 inhibitor scaffold identified in high-throughput screen. **(B)** Western blot depicting polyubiquitinated protein profile (K48 antibody) in worm lysate following treatment by DMSO control or p97 covalent inhibitor analogs (actin loading control). **(C)** Full structural activity relationship modifications of lead compound series outlining **R1** (800 series) and **R2** (700 series) modifications made to the depicted scaffold. Comparative IC_{50} values display the potency of each compound on the recombinant parasite (*Sm* p97) and human (*Hs* p97) enzyme. Compounds were tested from 100 µM – 1 nM. Values were calculated using Prism. EC_{50} values for benzoxazole propiolamide scaffold analogs on adult parasites determined by fraction of worms attached to tissue culture plate following 72 hours of drug treatment, refreshing media and drug every 24 hr, then allowing worms to remain in culture until D5 (50 µM - 10 nM) (negative; DMSO, positive; CB-5083). Values were calculated using Prism. Scale bar (A), 1,000 µm.

bioRxiv preprint doi: https://doi.org/10.1101/2025.03.14.643303; this version posted March 15, 2025. The copyright holder for this preprint (which was not certified by peer review) is the author/funder, who has granted bioRxiv a license to display the preprint in perpetuity. It is made available under aCC-BY 4.0 International license.

Supplemental Figure 10.

A

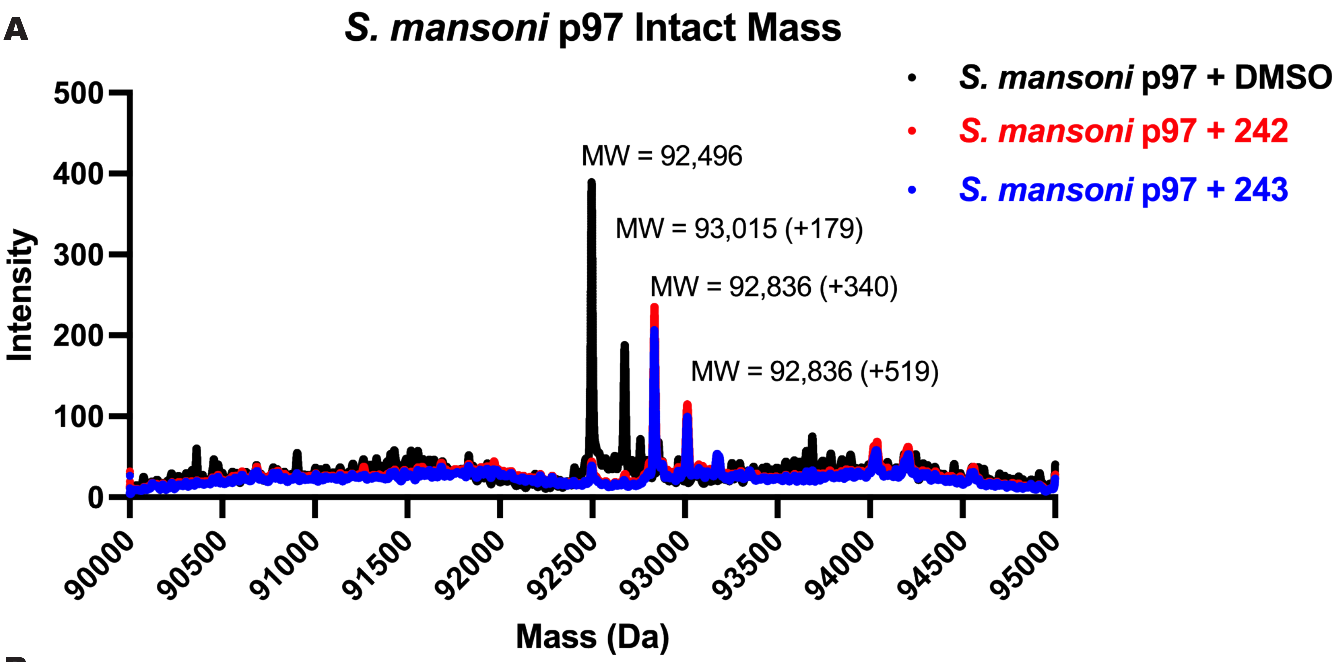

B

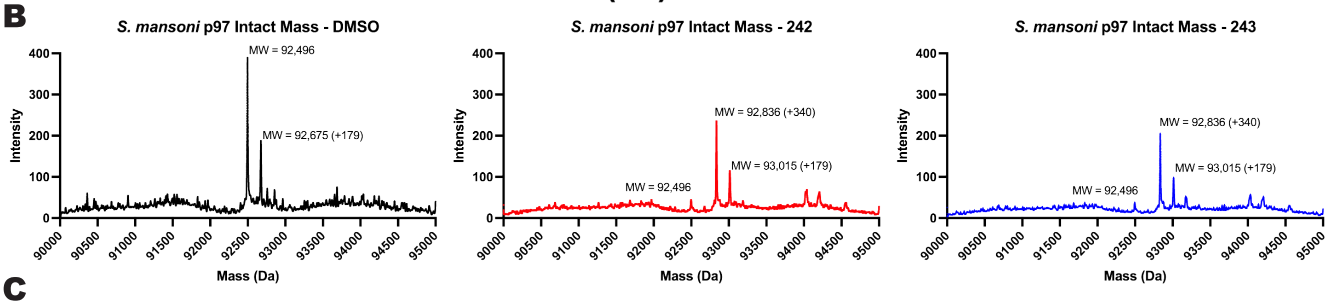

C

Confidence	Annotated Sequence	Modifications	# PSMs	Master Protein Accessions	Positions in Master Proteins	Master Protein Descriptions	Abundance DMSO	Abundance 242	Abundance 243
High	[K].ETVCVAIVDESCPDDKIR.[L]	1xCarbami-domethyl [C]; 1x242 [C]	5	Smp_018240.1	Smp_018240.1 [63-80]	transcript=Smp_018240.1 gene=Smp_018240		1.51E+05	2.02E+06
High	[K].VIETDPSPYCIVSPDTTIHTEGDPVK.[R]	1x242 [C10]	2	Smp_018240.1	Smp_018240.1 [162-187]	transcript=Smp_018240.1 gene=Smp_018240			5.08E+06
High	[K].VIETDPSPYCIVSPDTTIHTEGDPVKR.[E]	1x242 [C10]	3	Smp_018240.1	Smp_018240.1 [162-188]	transcript=Smp_018240.1 gene=Smp_018240			1.29E+07
High	[K].VIETDPSPYCIVSPDTTIHTEGDPVKREDEEEK.[L]	1x242 [C10]	1	Smp_018240.1	Smp_018240.1 [162-194]	transcript=Smp_018240.1 gene=Smp_018240	3.08E+05		5.90E+05
High	[K].REDEEEKLNEIGYDDIGGCR.[K]	1x242 [C19]	2	Smp_018240.1	Smp_018240.1 [188-207]	transcript=Smp_018240.1 gene=Smp_018240			3.26E+06
High	[K].REDEEEKLNEIGYDDIGGCRK.[Q]	1x242 [C19]	1	Smp_018240.1	Smp_018240.1 [188-208]	transcript=Smp_018240.1 gene=Smp_018240			2.02E+06
High	[R].EDEEEKLNEIGYDDIGGCR.[K]	1x242 [C18]	2	Smp_018240.1	Smp_018240.1 [189-207]	transcript=Smp_018240.1 gene=Smp_018240		1.10E+06	1.48E+07
High	[K].LNEIGYDDIGGCR.[K]	1x242 [C12]	1	Smp_018240.1	Smp_018240.1 [195-207]	transcript=Smp_018240.1 gene=Smp_018240			8.32E+06
High	[K].LNEIGYDDIGGCRK.[Q]	1x242 [C12]	1	Smp_018240.1	Smp_018240.1 [195-208]	transcript=Smp_018240.1 gene=Smp_018240			1.47E+07
High	[K].IANEAHGHVGADLASLCSEAALQQIR.[N]	1x242 [C17]	4	Smp_018240.1	Smp_018240.1 [396-421]	transcript=Smp_018240.1 gene=Smp_018240		1.78E+06	5.64E+07
High	[K].FGMTPSKGVLFYGPPGCGK.[T]	1x242 [C17]	1	Smp_018240.1	Smp_018240.1 [503-521]	transcript=Smp_018240.1 gene=Smp_018240		1.01E+05	1.53E+06
**High**	**[K].GVLFYGPPGCGK.[T]**	**1x242 [C10]**	**24**	**Smp_018240.1**	**Smp_018240.1 [510-521]**	**transcript=Smp_018240.1 gene=Smp_018240**		**8.13E+08**	**3.54E+09**
High	[R].QAAPCVLFFDELDSIAK.[A]	1x242 [C5]	2	Smp_018240.1	Smp_018240.1 [565-581]	transcript=Smp_018240.1 gene=Smp_018240		4.97E+06	5.59E+06
High	[K].ATQGFSGADLTEICQR.[A]	1x242 [C14]	1	Smp_018240.1	Smp_018240.1 [675-690]	transcript=Smp_018240.1 gene=Smp_018240			5.89E+06

bioRxiv preprint doi: https://doi.org/10.1101/2025.03.14.643303; this version posted March 15, 2025. The copyright holder for this preprint (which was not certified by peer review) is the author/funder, who has granted bioRxiv a license to display the preprint in perpetuity. It is made available under aCC-BY 4.0 International license.

Supplemental Figure 10. Mass spectrometry of covalent compounds in complex with schistosome p97

(A) Intact mass spectrum of recombinant *S. mansoni* p97 in solution with DMSO control (black) or covalent scaffold compounds (242 - red and 243 - blue). Major peaks consist of a single p97 monomer (~92.5 kDa) and an additional isoform (+179 Da). Mass shifts seen following incubation with either covalent inhibitor (339.35 Da) or DMSO. **(B)** Isolated mass spectrum of recombinant *S. mansoni* p97 in solution with DMSO control (black) or covalent scaffold compounds (242 - red and 243 - blue). **(C)** Table outlining detected peptides following incubation of schistosome p97 with covalent compound 242 or 243. Resulting reaction mixture was run on an SDS-PAGE, then the corresponding band was isolated and submitted for trypsin digest and peptide identification by LC-MS.

bioRxiv preprint doi: https://doi.org/10.1101/2025.03.14.643303; this version posted March 15, 2025. The copyright holder for this preprint (which was not certified by peer review) is the author/funder, who has granted bioRxiv a license to display the preprint in perpetuity. It is made available under aCC-BY 4.0 International license.

Supplemental Figure 11.

A

Compound	243	739	804	805	806	807	808	CB-5083
GI_{50} (µM)	5.6	0.85	0.93	1.2	1.5	1.1	2.6	0.31
IC_{50} (µM)	9.6	1.2	1.2	1.6	1.8	1.4	3.4	0.41

B

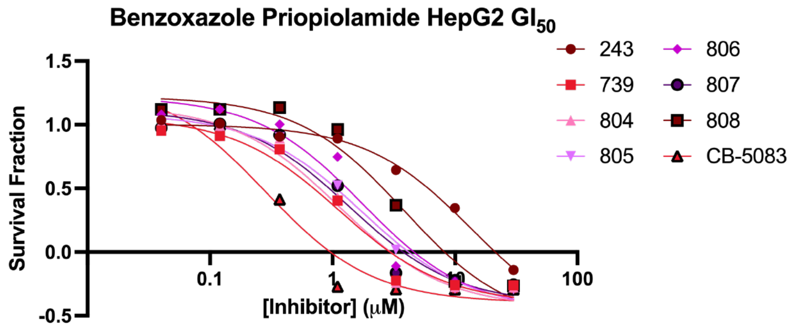

C

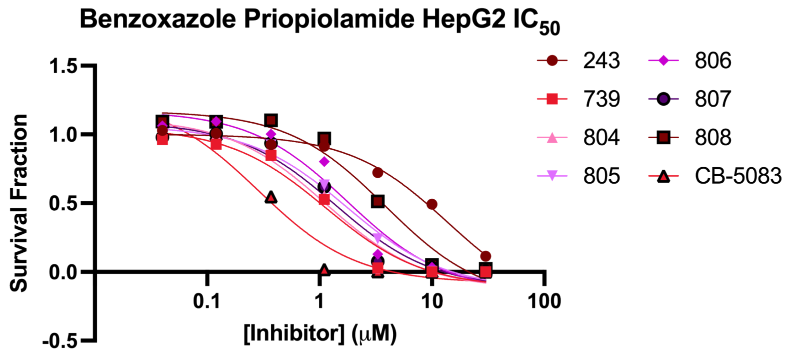

D

Compound	*S. mansoni* IC_{50} (µM)	*H. sapiens* IC_{50} (µM)	Fold Selectivity
242	1.4 ± 0.11	12 ± 0.31	8.6
243	2.1 ± 0.75	20 ± 0.54	9.5
244	>100	>100	NA
245	3.8 ± 0.19	>100	>30
732	>100	NA	NA
733	34 ± 1.6	43 ± 4.4	1.3
734	48 ± 2.6	83 ± 6.5	1.7
735	11 ± 0.47	7.3 ± 0.66	0.70
736	5.9 ± 1.3	6.7 ± 0.055	1.1
737	3.0 ± 0.41	13 ± 2.2	4.3
738	0.42 ± 0.057	0.78 ± 0.14	1.9
739	0.15 ± 0.049	0.32 ± 0.026	2.1
804	0.19 ± 0.063	0.14 ± 0.062	0.70
805	0.17 ± 0.092	0.14 ± 0.078	0.80
806	0.47 ± 0.17	0.51 ± 0.16	1.1
807	0.67 ± 0.17	0.49 ± 0.057	0.70
808	0.18 ± 0.026	0.41 ± 0.084	2.3
CB-5083	0.011 ± 2.1	0.022 ± 0.71	2.0

E

Compound	*S. mansoni* EC_{50} (µM)	HepG2 GI_{50} (µM)	HepG2 IC_{50} (µM)
242	9.9	NA	NA
243	3.5	5.6	9.6
244	25	NA	NA
245	12	NA	NA
732	1.3	NA	NA
733	3.2	NA	NA
734	7.0	NA	NA
735	4.2	NA	NA
736	12	NA	NA
737	22	NA	NA
738	3.5	NA	NA
739	1.3	0.85	1.2
804	0.76	0.93	1.2
805	2.0	1.2	1.6
806	3.2	1.5	1.8
807	1.8	1.1	1.4
808	2.4	2.6	3.4
CB-5083	0.36	0.31	0.41

bioRxiv preprint doi: https://doi.org/10.1101/2025.03.14.643303; this version posted March 15, 2025. The copyright holder for this preprint (which was not certified by peer review) is the author/funder, who has granted bioRxiv a license to display the preprint in perpetuity. It is made available under aCC-BY 4.0 International license.

Supplemental Figure 11. Covalent p97 inhibitor series HepG2 toxicity studies

(A) Table of GI_{50} (50% growth inhibition normalized to time = 0) and IC_{50} (50% growth inhibition relative to control) values for HepG2 cells treated by covalent p97 inhibitor scaffold compared to CB-5083 control. Values were plotted in excel and the 50% point was determined by interpolation. **(B)** Graphs visualized in Prism depicting GI_{50} and **(C)** IC_{50} curves for HepG2 cytotoxicity experiment following treatment by covalent analogs. Table comparing potencies on **(D)** p97 enzyme (schistosome and human) and **(E)** worms and human cells. Values were calculated in Prism.

bioRxiv preprint doi: https://doi.org/10.1101/2025.03.14.643303; this version posted March 15, 2025. The copyright holder for this preprint (which was not certified by peer review) is the author/funder, who has granted bioRxiv a license to display the preprint in perpetuity. It is made available under aCC-BY 4.0 International license.

500 **Supplemental Figure 12.**

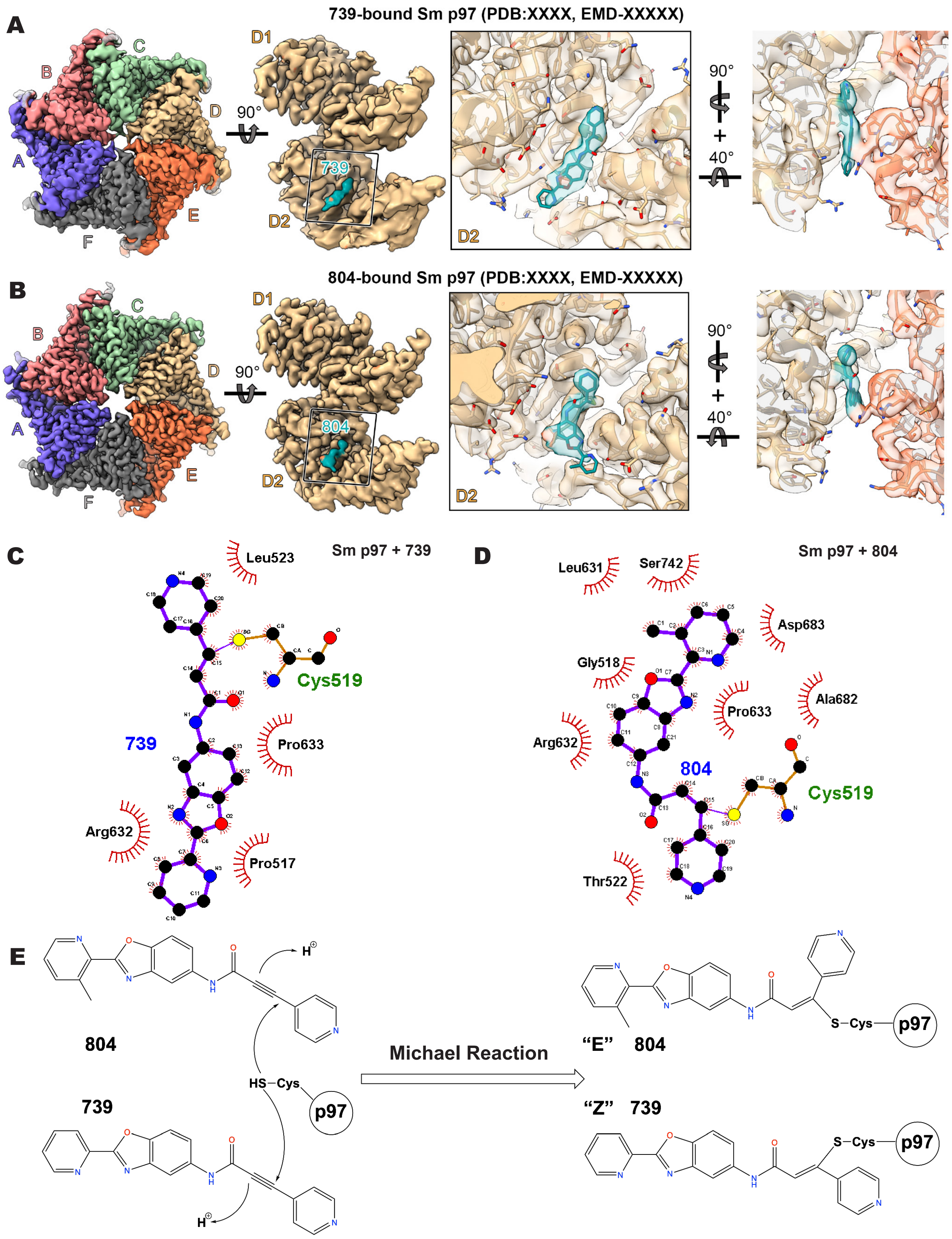

501

bioRxiv preprint doi: https://doi.org/10.1101/2025.03.14.643303; this version posted March 15, 2025. The copyright holder for this preprint (which was not certified by peer review) is the author/funder, who has granted bioRxiv a license to display the preprint in perpetuity. It is made available under aCC-BY 4.0 International license.

Supplemental Figure 12. Cryo-EM Structure of *S. mansoni* p97 - Compound 739 or 804 complex

(**A-B**) Cryo-EM map of the schistosome p97 bound to compound **(A)** 739 and **(B)** 804, colored by the final structures. Two zoom-in views of the D2 domain of *S. mansoni* p97 is shown on the right. (**C-D**) Ligplot of residues involved in binding to **(C)** 739 and **(D)** 804. **(E)** Schematic depicting Michael addition reaction conducted by the thiol side chain of Cys519 in the schistosome p97 resulting in "E-" and "Z-" olefin configuration liganded states for compounds 804 and 739, respectively.
